# Escalation of Pyrethroid Resistance in the Malaria Vector *Anopheles funestus* Induces a Loss of Efficacy of Piperonyl Butoxide–Based Insecticide-Treated Nets in Mozambique

**DOI:** 10.1093/infdis/jiz139

**Published:** 2019-03-29

**Authors:** Jacob M Riveron, Silvie Huijben, Williams Tchapga, Magellan Tchouakui, Murielle J Wondji, Micareme Tchoupo, Helen Irving, Nelson Cuamba, Mara Maquina, Krijn Paaijmans, Charles S Wondji

**Affiliations:** 1 Vector Biology Department, Liverpool School of Tropical Medicine (LSTM), United Kingdom; 2 Centre for Research in Infectious Diseases (CRID), Yaoundé, Cameroon; 3 Center for Evolution and Medicine, School of Life Sciences, Arizona State University, Tempe; 4 ISGlobal, Barcelona, Spain; 5 Instituto Nacional de Saúde, Maputo; 6 Centro de Investigação em Saúde da Manhiça, Mozambique

**Keywords:** malaria, insecticide resistance, vector control, *An. funestus*, Mozambique, long-lasting insecticidal nets, metabolic resistance, cytochrome P450

## Abstract

**Background:**

Insecticide resistance poses a serious threat to insecticide-based interventions in Africa. There is a fear that resistance escalation could jeopardize malaria control efforts. Monitoring of cases of aggravation of resistance intensity and its impact on the efficacy of control tools is crucial to predict consequences of resistance.

**Methods:**

The resistance levels of an *Anopheles funestus* population from Palmeira, southern Mozambique, were characterized and their impact on the efficacy of various insecticide-treated nets established.

**Results:**

A dramatic loss of efficacy of all long-lasting insecticidal nets (LLINs), including piperonyl butoxide (PBO)–based nets (Olyset Plus), was observed. This *An. funestus* population consistently (2016, 2017, and 2018) exhibited a high degree of pyrethroid resistance. Molecular analyses revealed that this resistance escalation was associated with a massive overexpression of the duplicated cytochrome P450 genes *CYP6P9a* and *CYP6P9b*, and also the fixation of the resistance CYP6P9a_R allele in this population in 2016 (100%) in contrast to 2002 (5%). However, the low recovery of susceptibility after PBO synergist assay suggests that other resistance mechanisms could be involved.

**Conclusions:**

The loss of efficacy of pyrethroid-based LLINs with and without PBO is a concern for the effectiveness of insecticide-based interventions, and action should be taken to prevent the spread of such super-resistance.

## Teaser

An extensive loss of efficacy of all pyrethroid-based Insecticide-treated bednets, including PBO-based bednets is recorded in a highly pyrethroid resistant population of the malaria vector *An. funestus* (Mozambique) exhibiting high expression of cytochrome P450 genes with fixation of CYP6P9a_R allele.

Malaria burden remains high in Africa [1], despite recent progress achieved mainly through insecticide-based interventions such as long-lasting insecticidal nets (LLINs) and indoor residual spraying (IRS) [2, 3]. Increasing reports of resistance to major insecticide classes is a worrying concern for the continued effectiveness of insecticide-based control tools. The resistance to pyrethroids is particularly problematic, as it is the main insecticide class approved for LLIN impregnation, as well as the most common insecticide class used in IRS [4]. Therefore, devastating consequences are predicted for malaria control if pyrethroid efficacy is lost, as highlighted by the World Health Organization (WHO) [5]. However, there is currently an intense debate, with opposite results often published about the impact of insecticide resistance on the effectiveness of insecticide-based interventions [6, 7]. This contrast is highlighted by the differences observed between a multicountry study showing a lack of impact of pyrethroid resistance on malaria transmission [6], compared with a field trial in Tanzania, which supported that pyrethroid resistance was reducing the effectiveness of pyrethroid-only LLINs and impacting malaria transmission [7]. Among other factors, it is possible that this discrepancy is associated with the variation of the strength of resistance in respective populations studied. Indeed, it is acknowledged that increasing resistance levels (resistance ratio) are more likely to lead to control failure than standard resistance levels [8, 9]. This highlights the crucial need to monitor field populations for evidence of resistance escalation and to measure the potential impacts of resistance escalation on the efficacy of insecticide-based tools including LLINs. However, limited studies have been performed on the escalation of resistance in field populations of malaria vectors in Africa. A study in Burkina Faso (West Africa) revealed that increased resistance in *Anopheles gambiae* negatively impacted the efficacy of pyrethroid-only nets [10]. Similarly, a loss of efficacy of pyrethroid-only nets was observed in a population of *Anopheles funestus* sensu stricto (s.s.) in southern Mozambique (southern Africa) [11], previously shown to be highly resistant to pyrethroid [11, 12], suggesting that this population could be ideal to monitor the increase in resistance levels and its consequences.

Pyrethroid resistance in *An. funestus* is widespread throughout Mozambique, notably in the south where mosquitoes have been shown to survive 3 hours of exposure to pyrethroids in WHO bioassays [12–16]. This resistance is driven by metabolic resistance mediated by overexpression of cytochrome P450 (CYP), including 2 duplicated CYPs, *CYP6P9a* and *CYP6P9b* [17, 18]. The recent detection of a DNA-based marker for the *CYP6P9a* resistance allele revealed that the CYP6P9a_R frequency was elevated in southern Mozambique [19]. In contrast, to date, no knockdown resistance (kdr) mutation in the voltage-gated sodium channel gene has been reported in *An. funestus* s.s. Africa-wide [17, 20, 21]. A new generation of LLINs, combining a pyrethroid with the synergist piperonyl butoxide (PBO), has been designed by manufacturers to overcome this growing problem of pyrethroid resistance. PBO inhibits the action of the CYPs [22, 23], enhancing the effect of pyrethroids on resistant kdr-free mosquitoes [24–26]. The impact of increased resistance levels in southern Mozambique remains unelucidated on the efficacy of PBO-based nets. It remains also unknown if the escalation of resistance is associated with overexpression of metabolic resistance genes such as *CYP6P9a/b* and if such increased expression of CYP genes could reduce the inhibition effect of PBO and reduce the efficacy of PBO-based nets.

To fill this gap and facilitate the design of resistance management strategies, we extensively investigated the resistance profile and resistance mechanisms of one highly resistant population of *An. funestus* s.s. in southern Mozambique (Palmeira).

## METHODS

### Mosquito Collection

Indoor female *Anopheles* mosquitoes were collected in the village of Palmeira (25° 15′ 19′′ S; 32° 52′ 22′′ E), Manhiça district, Maputo province (southern Mozambique) near the Incomati river. The majority of inhabitants are farmers (sugar cane, rice) from the Xichangana and Xironga communities. The collections were performed during 4–5 days in 3 consecutive years (August 2016, April 2017, and January 2018) using electric aspirators. *Anopheles funestus* s.s. is the primary malaria vector in this area [27]. An *Anopheles funestus* sample collected in 2002 [28] was used for comparative genotyping of the *CYP6P9a* resistance allele. Most of the households have LLINs (Olyset and PermaNet 2.0) impregnated only with pyrethroids, whereas IRS with dichlorodiphenyltrichloroethane (DDT) is also applied [27].

Collected *Anopheles* female mosquitoes (blood-fed, gravid and half-gravid) were morphologically identified as belonging to *An. funestus* group or *An. gambiae* complex according to morphological keys [29]. *Anopheles funestus* sensu lato (s.l.) females were kept in cages until they became fully gravid, and subsequently, forced to lay eggs in separate 1.5-mL microcentrifuge tubes and larvae reared to adults as previously described [30]. Seventy *An. funestus* s.l. females collected in April 2017 were bisected into head plus thorax and abdomen and kept individually. Genomic DNA (gDNA) from these mosquitoes were extracted using the Livak method [31], followed by a cocktail polymerase chain reaction (PCR) as previously described [32] for species identification with *An. funestus* group. The internal transcribed spacer 2 (ITS2) was sequenced for samples that failed to amplify.

### *Plasmodium* Infection Rates

A TaqMan assay was used to screen for *Plasmodium falciparum* and for *Plasmodium ovale*, *Plasmodium vivax*, *and Plasmodium malariae* (ovm) in 57 heads plus thoraxes gDNA (sporozoite) from 2017 field collected (F_0_) *An. funestus* s.s. females as previously described [33, 34]. Subsequently, a nested PCR [35] was also performed to validate all of the *Plasmodium*-positive samples.

### Insecticide-Treated Bed Net Efficacy Assays

Following the WHO guidelines for cone bioassays [36], the effectiveness of the following LLINs was estimated for Olyset Net (permethrin 2%) and Olyset Plus net roof (permethrin 2% plus PBO 1% in the roof); and PermaNet 2.0 (deltamethrin 0.18%) and PermaNet 3.0 side (deltamethrin 0.28%). An untreated mosquito net was used as a control. Five replicates of 10 first generation (F_1_) 2- to 5-day-old females were placed in plastic cones enclosed with the mosquito net during 3 minutes of exposure. Mosquitoes were then placed in small holding paper cups with cotton soaked in 10% sugar solution. Mortality was determined 24 hours later in 2016 and 2018, and every 24 hours, until 5 days in 2017. The efficacy of the LLINs was confirmed using the *An. gambiae* susceptible laboratory strain Kisumu (2016 and 2017) and the *An. funestus* susceptible FANG strain (2018).

### Insecticide Susceptibility Assays

The insecticide resistance profiles of *An. funestus* s.s. were assessed using the WHO tube bioassays [37]. *Anopheles funestus* s.s. mosquitoes collected in 2016 were tested to the pyrethroids type I permethrin (0.75%) and type II deltamethrin (0.05%), the organochlorine DDT (4%), and the carbamate bendiocarb (0.1%). Mosquitoes collected in 2017 were additionally tested with the pyrethroid derivative etofenprox (0.05%) and the organophosphate malathion (5%). Assays were performed at 25°C ± 1°C (± SD) and 70%–80% relative humidity. At least 3 replicates of 20–25 F_1_ female and male mosquitoes 2–5 days old were exposed separately to insecticide-impregnated papers for 1 hour and afterward transferred to a holding tube provided with cotton soaked in 10% sugar solution. Mortality was determined 24 hours later. Control tubes using carrier oil–impregnated papers were performed for each bioassay. Synergist assays with PBO (inhibitor of CYPs) was performed as previously described [38].

Additionally, due to the extremely high resistance to permethrin, an insecticide commonly used in LLINs, the intensity of resistance was assessed by exposing 3 replicates of 20–25 F_1_ female mosquitoes for 90, 120, and 180 minutes to permethrin in WHO tube bioassays as described above.

### Transcription Profile of Resistance Genes in *An. funestus* s.s

Total RNA was extracted from 3 batches of 10 adult 2- to 5-day-old F_1_ female *An. funestus* s.s. nonexposed to insecticides and similarly from the susceptible laboratory strain FANG, as previously described [17]. The transcription patterns of the duplicated CYP genes *CYP6P9a* and *CYP6P9b*, major pyrethroid resistance genes in this region [17, 39], plus the DDT/permethrin-resistant gene-related glutathione-s-transferase epsilon 2 (*GSTe2*) [40], were assessed by a quantitative reverse-transcription polymerase chain reaction (qRT-PCR) assay, as previously described [17, 41]. The relative expression was calculated individually according to the 2-ΔΔCT method [42] and compared to that previously published in southern Africa (Malawi), West Africa (Ghana and Benin), East Africa (Uganda), and Central Africa (Cameroon) [43].

### Genotyping of the CYP6P9a_R Pyrethroid Resistance Allele

A recently designed PCR–restriction fragment length polymorphism (RFLP) assay [19] was used to genotype the CYP6P9a_R allele in Palmeira in 2016 and 2017 but also in 2002 to assess potential link between the aggravation of resistance and this allele.

### Genotyping of Other Resistance Markers in *An. funestus* s.s

The presence of other *An. funestus* s.s. resistance markers was assessed including N485I-Ace-1 (bendiocarb), A296S-RDL (dieldrin), and L119F-GSTe2 (DDT/permethrin) using TaqMan assays, as previously described [44, 45] (N485I-Ace-1 and A296S-RDL) and an allele-specific PCR assay (L119F-GSTe2) [46].

## RESULTS

### Species Identification

A total of 750, 1100, and 425 F_0_ female mosquitoes were sampled in 2016, 2017, and 2018, respectively, with 40%–50% blood-fed, half-gravid or fully gravid. A total of 243, 360, and 185 females laid eggs in 2016, 2017, and 2018, respectively, with 120, 250, and 92 hatching. From 96 (2016), 70 (2017), and 50 (2018) F_0_ female *An. funestus* s.l. molecularly assessed by PCR, 90, 57, and 50 females were identified as *An. funestus* s.s., respectively, whereas 6 (2016) and 13 (2017) failed to amplify. Subsequently, through sequencing of the ITS2, the 13 samples (2017) were also identified as *An. funestus* s.s.

### *Plasmodium* Sporozoite Infection Rate

The *Plasmodium* sporozoite infection rate in *An. funestus* s.s. (2017) was 5.3% (3/57). Two *An. funestus* s.s. females were infected *with P. falciparum* sporozoites (2/57 [3.5%]), and one was infected with ovm (1/57 [1.8%]). A nested PCR confirmed all of the positive infected mosquitoes and determined that the ovm-positive sample was infected with *P. malariae*.

### Insecticide-Treated Bed Net Efficiency

**Figure 1.**
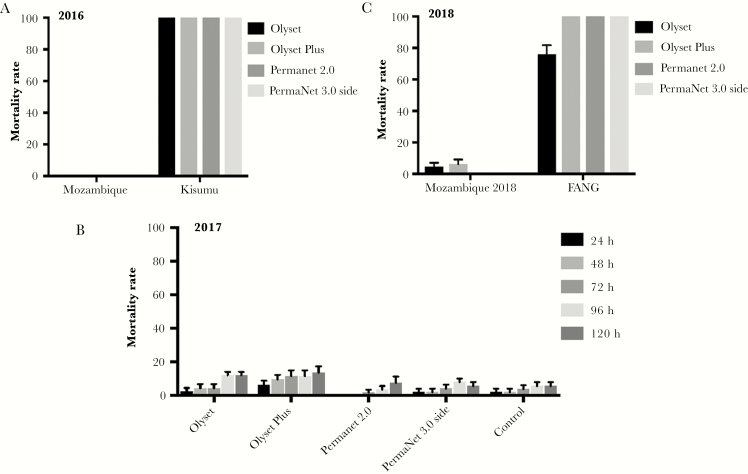
Bioefficacy of different commercial long-lasting insecticidal nets against *Anopheles funestus* sensu stricto in Palmeira, Mozambique, in 2016 (*A*), 2017 (*B*), and 2018 (*C*). Error bars represent standard error of the mean.

No mortality was recorded following 3-minute exposure for all LLINs tested including the Olyset Plus PBO-based net against F_1_ females collected in 2016, suggesting an extensive loss of efficacy of these nets. However, the same nets induced a total mortality against the control Kisumu susceptible *An. gambiae* mosquitoes (Figure 1A). To confirm these results, a new batch of LLINs was tested against another sample of *An. funestus* s.s. collected in 2017, revealing similar loss of efficacy with mortalities <6.2% after 24 hours. Due to this exceptional loss of efficiency, the mortality was also monitored at 48, 72, 96, and 120 hours after exposure in 2017 (Figure 1B). After 120 hours, similar low mortalities (between 5.7% and 7.5%) were recorded for both LLINs impregnated with deltamethrin (PermaNet 2.0 and 3.0 [side]) and control, whereas the 2 LLINs impregnated with permethrin (Olyset Net and Olyset Plus) presented a slightly higher mortality (12% and 13.6%, respectively). A similar loss of efficacy was observed in 2018 (Figure 1C) in contrast to the high mortality in FANG, the *An. funestus* susceptible laboratory strain. These results indicate a surprisingly extensive loss of action of the PBO in the PBO-based LLINs (Olyset Plus) against this *An. funestus* population. Mortality rate with the control net was 0% in 2016 and 2017 and 2% in 2018.

### Insecticide Susceptibility Assays

**Figure 2.**
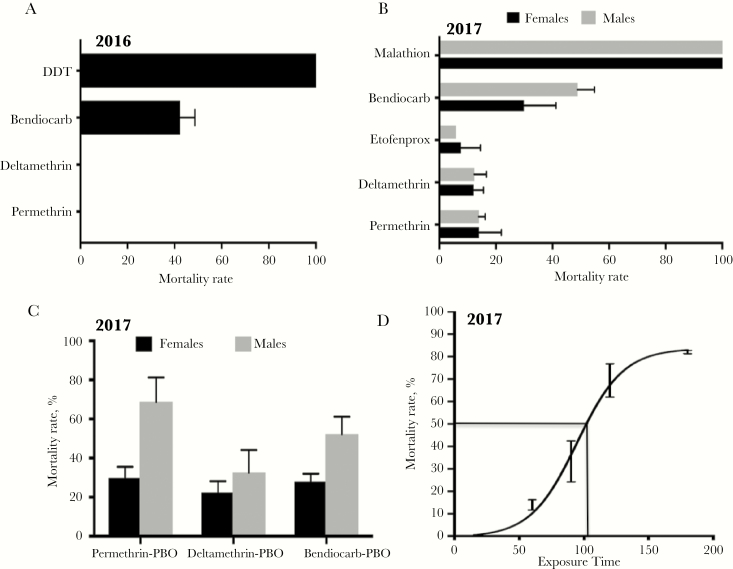
Susceptibility profiles of an *Anopheles funestus* population from Palmeira, southern Mozambique. *A*, Susceptibility profile in 2016 for females only. *B*, Susceptibility profile in 2017 for both males and females. *C*, Synergist assay with piperonyl butoxide (n = 4). *D*, Time-point mortality rates for permethrin with estimation of the median lethal time (LT_50_) at 1 hour 45 minutes. Error bars represent standard error of the mean. Abbreviations: DDT, dichlorodiphenyltrichloroethane; PBO, piperonyl butoxide.

F_1_ females from both collections exhibited an extremely high resistance to permethrin (pyrethroid type I; used in Olyset nets) and deltamethrin (pyrethroid type II; used in PermaNet nets), supporting the observed loss of efficiency of LLINs (Figure 2A and 2B). F_1_ females collected in 2016 showed no mortality after 24 hours of exposure to both pyrethroids tested (permethrin and deltamethrin) (Figure 2A), whereas F_1_ females collected in 2017 showed 13.9% ± 2.4% and 12.3% ± 4.3% mortality to permethrin and deltamethrin, respectively (Figure 2B). F_1_ females collected in 2017 also presented high resistance to the pyrethroid derivative etofenprox (5.9% ± 0.2% mortality) and to the carbamate bendiocarb, with mortalities of 42.3% ± 6.3% and 29.8% ± 11.4% in 2016 and 2017, respectively (Figure 2B). However, it consistently showed a full susceptibility to the organochlorine DDT and the organophosphate malathion with 100% mortality rates. Male mosquitoes (2017) exhibited similar a resistance profile (Figure 2B).

Synergist assays performed in 2017 with PBO revealed only a moderate recovery of susceptibility after exposure to permethrin and deltamethrin (permethrin: no PBO preexposure, 13.9% ± 2.4% mortality vs PBO preexposure, 29.7% ± 5.8%, *P* = .065; deltamethrin: no PBO preexposure, 12.3% ± 4.3% vs PBO preexposure, 22.3% ± 15.9%, *P *= .24) (Figure 2C). Tests with bendiocarb also revealed a lack of impact of PBO preexposure with no difference in mortality with PBO exposure (27.9 ± 4% mortality, *P = *.09) vs 29.8% ± 11% without exposure. No mortality was observed in control mosquitoes exposed to the synergist PBO only.

Due to the high resistance observed to pyrethroids, WHO tube bioassays with F_1_ females collected in 2017 and exposure times of 90, 120, and 180 minutes to permethrin were also performed (Figure 2D). A constant increase in mortality was observed proportional to the time of the permethrin exposure, with median lethal time (LT_50_) estimated at 1 hour 45 minutes (95% confidence interval, 1 hour 37 minutes to 1 hour 51 minutes).

### Transcription Profile of Resistance Genes in *An. funestus* s.s

**Figure 3.**
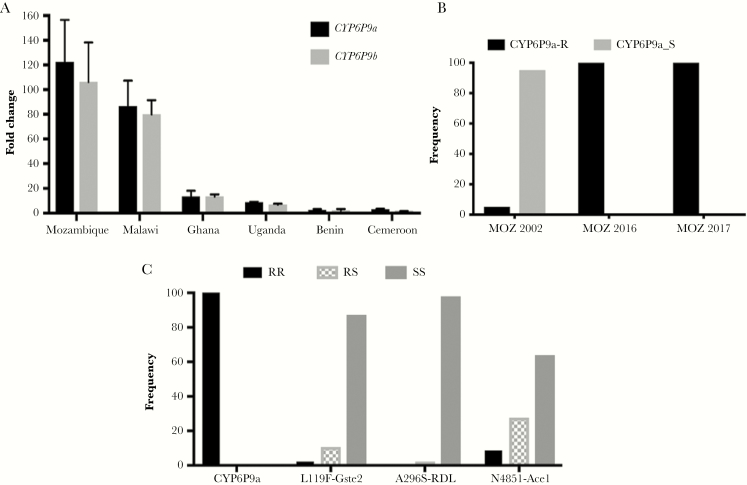
Exploration of the molecular basis of the escalation of pyrethroid resistance in *Anopheles funestus*. *A*, Comparative gene expression of the cytochrome P450 genes *CYP6P9a* and *CYP6P9b* in Mozambique comparatively to other African regions. Error bars represent standard error of the mean. *B*, Allele frequency of the *CYP6P9a* pyrethroid resistance marker in southern Mozambique populations from 2002, 2016, and 2017, showing a fixation of the CYP6P9a_R allele. *C*, Distribution of the genotypes of resistance markers in Palmeira including CYP6P9a_R, L119F-GSTe2, N485I-Ace-1, and A296S-RDL. Abbreviations: CYP, cytochrome P450; MOZ, Mozambique.

Transcription analysis of the duplicated CYP genes *CYP6P9a* and *CYP6P9b*, known to confer pyrethroid resistance in *An. funestus* [17, 39], reveals a high up-regulation of *CYP6P9a* (fold change [FC], 122.4 ± 34) and *CYP6P9b* (FC, 106.2 ± 32) from southern Mozambique compared to the susceptible FANG strain (*P* < .001). This overexpression is higher than in Malawi (FC, 85.5 ± 20.7 and 79.9 ± 11.6 for *CYP6P9a* and *CYP6P9b*, respectively) (Figure 3A), although not statistically significant (*P* > .05). A greater contrast is observed with other African regions where the overexpression of these 2 genes is significantly much lower (*P* < .001) (Figure 3A). In contrast, *GSTe2*, conferring DDT/permethrin resistance in West/Central Africa [40], is not significantly upregulated in Mozambique (FC, 1.1 ± 0.4; *P* = .84).

### Frequency of the CYP6P9a_R Allele

Genotyping of 50 (2016) and 57 (2017) F_0_ females using the novel PCR-RFLP assay [19] revealed that this resistance allele for CYP-mediated metabolic resistance is fixed in Palmeira with 100% of the RR genotype detected in both samples. This contrasts with 2002 (35 females) where the CYP6P9a_R allele was only recorded at 5% (Figure 3B) (χ^2 ^= 1900, *P* = .0) suggesting that, beside other factors including increased overexpression of CYPs, the escalation of resistance could have been associated with the fixation of the CYP6P9a_R allele in field population.

### Frequency of Other Resistance Alleles

The frequency of the 119F-GSTe2 resistant allele was very low (7.4%) (1RR, 6RS, and 47SS; Figure 3C). This is the first detection of this resistance marker in southern Africa as it was completely absent from samples collected in 2010 [40].

The frequency of the A296S-RDL mutation conferring dieldrin resistance [47] was very low (0.9%) (0RR, 1RS, and 56SS; Figure 3C). However, this is its first report in southern Africa.

The N485I mutation in the acetylcholinesterase 1 gene associated with bendiocarb resistance [45] was detected at a frequency of 23.9% (5 RR-485I, 18 RS-N485I, and 34 SS-N485; Figure 3C).

## Discussion

Assessing the dynamic of resistance to insecticides in major malaria vectors and its impact on the effectiveness of control tools is a key prerequisite for the implementation of suitable strategies to manage the growing challenge from insecticide resistance in malaria control.

This study revealed a complete loss in efficacy of the 2 most common commercial LLINs used across Africa, Olyset Net and PermaNet 2.0, against *An. funestus* s.s. from southern Mozambique, confirming the extensive loss reported for these nets in 2015 in the same region [11]. A similar extensive loss was also reported in Malawi (<5% mortality) [44] and in the Democratic Republic of Congo (DRC; <35% mortality) [38]. This loss in efficacy in Palmeira is further supported by the WHO insecticide susceptibility assays results, showing a high resistance of this population to permethrin and deltamethrin, the pyrethroid compounds used to impregnate these nets. This high pyrethroid resistance is in line with previous reports in this region [11, 12].

More alarmingly, this study also reported the extensive loss in efficacy of the new generation of PBO-based nets, prior to the implementation of PBO-based bed nets in the area. This is the first report of such loss of efficacy of these new-generation nets against *An. funestus*, as higher mortality rates (>80%) have so far been observed when testing PBO-based nets (Olyset Plus) against other pyrethroid-resistant populations including in Malawi [44] and DRC [38]. The extensive ability of mosquitoes to survive exposure to PBO-based nets in this population also differs significantly from results in *An. gambiae* for which highly resistant populations have shown mortality of around 40% such as in Burkina Faso [10] and DRC [38]. However, the PermaNet 3.0 Top (containing PBO) was not analyzed. Nevertheless, the low mortality with PermaNet 3.0 (side) here contrary to the higher mortality rate (88%) observed in DRC also suggests a loss of efficacy of PermaNet 3.0. In this study, we did not analyze the chemical content of the nets (using high-performance liquid chromatography) to confirm that the quantity of PBO or pyrethroids on the nets tested are those stipulated by the manufacturers. Such work will need to be done in the future as one cannot rule out some loss of the active ingredients in the nets. However, when checking these same nets against the susceptible laboratory strains (Kisumu and FANG), we observed a total mortality supporting the loss of efficacy against the field-resistant *An. funestus* populations.

The low mortality against PBO-based nets of the *An. funestus* population of southern Mozambique is further remarkable, as this population does not possess the kdr mutation [21, 28], contrary to the *An. gambiae* VK7 population from Burkina Faso. The lack of kdr in this Mozambican population is further supported by a total susceptibility of this population to DDT, a chemical that also targets the sodium channel gene. Previous studies and the qRT-PCR performed here indicate a predominant role of metabolic resistance in this *An. funestus* population primarily driven by the duplicated CYP genes *CYP6P9a* and *CYP6P9b* [17]. It is possible that the reduced efficacy of all bed nets, including PBO-based, is partly due to a dramatic overexpression of these CYPs that could allow mosquitoes to withstand exposure to pyrethroids even with the amount of PBO present in the nets. This is supported by the highest level of expression observed in this study in Mozambique for *CYP6P9a* and *CYP6P9b* compared even with another southern African population in Malawi, but even more when compared with other regions in East, Central, and West Africa. The role of *CYP6P9a/b* is further supported by the observation that the resistant allele of *CYP6P9a*, which has been demonstrated to have a higher catalytic efficiency in metabolizing pyrethroids than other alleles [39], has now been driven to fixation in this population whereas it was only present at 5% back in 2002. Such selection was recently shown to be driven by scale-up of insecticide-based interventions in the region, including pyrethroid-based IRS and LLINs [28]. Therefore, it is possible that the concentration of PBO in the LLINs is not sufficient to inhibit the action of the highly overexpressed and metabolically efficient *CYP6P9a*/*CYP6P9b* alleles in resistant mosquitoes from Palmeira.

However, the low recovery of mortality observed here after exposure to PBO during synergist assay, besides massive overexpression of CYPs such as *CYP6P9a/b*, could also be due to other mechanisms that are present in this *An. funestus* population. A potential role of the reduced penetration due to thickening of the mosquito’s cuticle was previously suggested in the laboratory strain FUMOZ-R, which originated from southern Mozambique [48]. Such a mechanism could also be acting in field populations of southern Mozambique, contributing to the high resistance to pyrethroids and the loss of efficacy observed in combination with high overexpression of highly efficient CYP enzymes. However, future studies are needed to elucidate the molecular basis of this resistance escalation that is inducing such loss of efficacy of all LLINs, including PBO-based nets.

The results of this study support the IRS interventions in southern Mozambique particularly based on organophosphates. Furthermore, the full susceptibility to DDT supports the lack of overexpression of the *GSTe2* gene in this population, contrary to what has been shown in West and Central Africa [19, 40]. However, this study detected for the first time in southern Africa the resistant allele, 119F-GSTe2, frequently present in West and Central Africa [40]. The increased frequency of this allele in southern Africa, in combination with high overexpression of CYP genes, could lead to super-resistance to pyrethroids and also DDT.

## Conclusions

The loss in efficiency of new-generation PBO-based LLINs against *An. funestus* s.s. reported here represents a serious challenge for its future implementation in southern Mozambique. The spread of the molecular mechanisms that confer this “resistance” to the synergistic effects of PBO to other vector populations is an even more worrying concern. This study highlights the urgent need to investigate the causes of the loss in efficacy of PBO-based nets and to monitor the spread of such operationally significant resistance in other mosquito populations and assess its impact on malaria transmission. Furthermore, efficacy of PBO-based nets should be assessed prior to the rolling out of these nets in Mozambique.

## Notes

Notes

***Author contributions.*** C. S. W. designed the study. J. M. R., S. H., and K. P. P. supervised the field work. N. C. and M. M. contributed to field work. J. M. R., W. T., and M. T. performed the resistance testing in the insectary. M. J. W., M. T., J. M. R., H. I., and C. S. W. performed the molecular experiments. J. M. R. and C. S. W. analyzed the data. J. M. R. and C. S.W. wrote the manuscript with contribution from all the authors. All authors read and approved the final manuscript.

***Acknowledgments.*** ISGlobal is a member of the CERCA Programme, Generalitat de Catalunya.

***Financial support.*** This work was supported by a Wellcome Trust Senior Research Fellowship in Biomedical Sciences to C. S. W. (award number 101893/Z/13/Z). S. H. was supported by the Society in Science–Branco Weiss Fellowship. K. P. was supported by the Bill & Melinda Gates Foundation and the Obra Social “la Caixa” Partnership for the Elimination of Malaria in southern Mozambique (award number OPP1115265).

***Potential conflicts of interest***. All authors: No reported conflicts of interest. All authors have submitted the ICMJE Form for Disclosure of Potential Conflicts of Interest. Conflicts that the editors consider relevant to the content of the manuscript have been disclosed.
